# Isolated Dizziness as an Atypical Presentation of Ethmoid Sinus Mucocele: A Case Report

**DOI:** 10.7759/cureus.89024

**Published:** 2025-07-29

**Authors:** Karim K Kalout, Wendy E Saliba, Samer R Nasr

**Affiliations:** 1 Cardiology, University of Balamand, Beirut, LBN; 2 Cardiology, Mount Lebanon Hospital, Beirut, LBN; 3 Medicine, University of Balamand, Beirut, LBN

**Keywords:** anterior ethmoid sinus, dizziness, endoscopic sinus surgery, ethmoid mucocele, orbital compression, paranasal sinus lesion, sinusitis complications, skull base lesion, subdural hematoma, vestibular symptoms

## Abstract

Paranasal sinus mucoceles are benign, expansile lesions that most commonly affect the frontal sinus with less frequent ethmoid sinus involvement. The most common presentation of these lesions is nasal or orbital symptoms due to their anatomical proximity to critical structures. Vestibular symptoms such as dizziness are exceedingly rare and unreported in the literature as a primary presentation of ethmoid mucoceles. We describe the case of a 77-year-old male patient with a history of well-controlled hypertension and hyperlipidemia who experienced a fall at home due to a sudden onset of dizziness, without losing consciousness. He reported persistent dizziness after the fall for three consecutive days, which prompted him to seek medical attention. Physical examination was unremarkable. Imaging performed revealed a chronic subdural hematoma and a left anterior ethmoid mucocele, which was compressing the medial orbit. He underwent endoscopic sinus surgery, which uncapped and drained the left ethmoid mucocele, after which the patient’s clinical condition was noted to have improved. This anterior ethmoid mucocele presenting as isolated dizziness is indeed rare and unusual. This case illustrates the need to consider paranasal sinus disease in the differential diagnosis of peripheral vestibular disorders in the elderly. Prompt imaging combined with timely surgery may enhance outcomes and further avert complications.

## Introduction

A mucocele is classified as a benign cystic lesion that is characterized by a mucus-producing epithelial lining of cells [1,2]. It occurs due to a blockage of the excretory duct, which slowly fills the cyst with mucus. The cyst can slowly enlarge, potentially compressing nearby structures [3,4]. Paranasal sinus mucoceles are rarely seen in ethmoid and maxillary sinuses and most commonly involve the frontal sinus (60-89%) [5]. Ethmoid mucoceles are further classified according to their location as anterior or posterior [6], and this results in distinct clinical symptomatology based on the structures involved [7]. The proximity of these mucoceles to the orbits and skull base predisposes the patient to significant unwanted complications and morbidities [4].

Herein, we report an unusual case of a male with a relatively large anterior ethmoid mucocele who presented with symptoms of isolated dizziness, which was successfully treated by endoscopic resection.

## Case presentation

A 77-year-old male with a known history of well-controlled hypertension and hyperlipidemia presented with persistent isolated dizziness that began three days before presentation. The dizziness occurred suddenly at home, which caused him to fall in the bathroom. He reports no loss of consciousness but continued to feel dizzy for the subsequent three consecutive days before seeking medical attention.

The patient reports feeling fatigued, with a decreased appetite, and a cough that is productive of a yellow-green sputum and rhinorrhea during the same period. He denied fever, chills, seizures, diplopia, or vision change, though he has a baseline of chronic visual impairment. The patient has no nasal obstruction or facial pain, no otalgia, but acknowledges a pre-existing mild hearing loss. He has a history of recurrent sinusitis but has never had sinus surgery before. He is a one-pack-year smoker and drinks alcohol socially.

**Figure 1:**
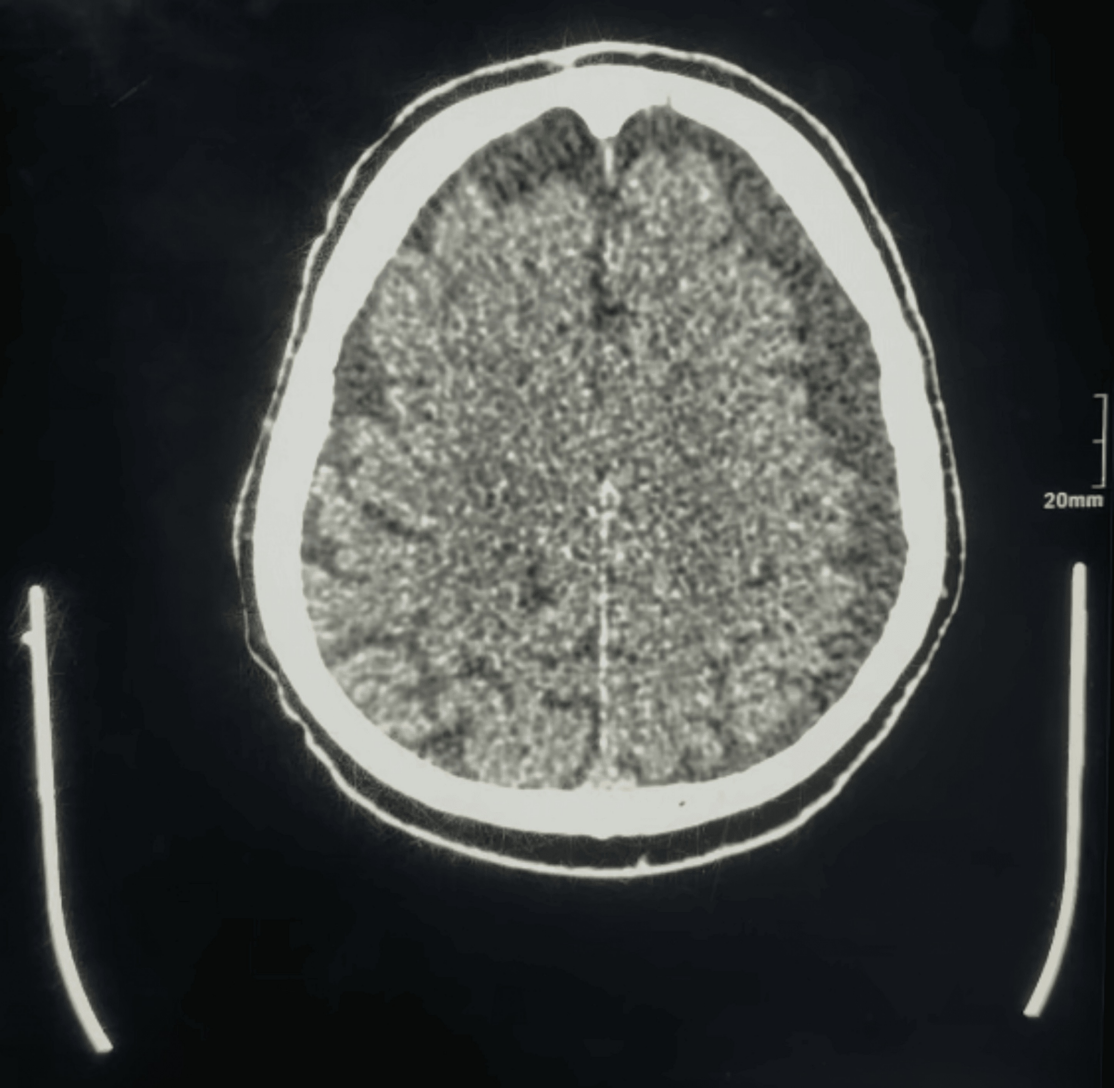
Preoperative sinus CT finding showing the left subdural hygroma CT: computed tomography

**Figure 2:**
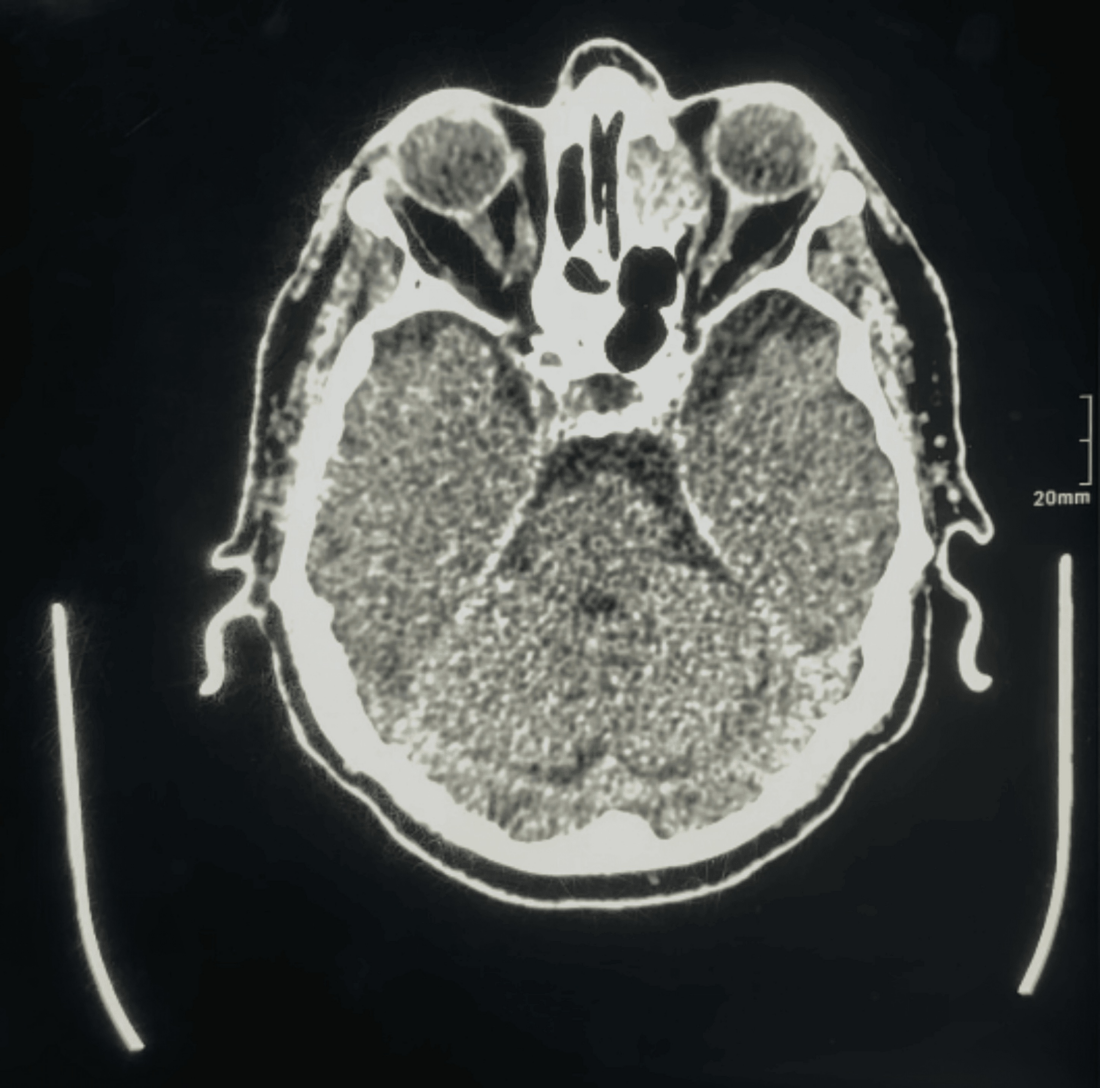
Preoperative sinus CT finding Opacification of the left anterior ethmoid air cell shows a bulge with underlying bone remodeling, lysis, and compression over the medial wall of the left orbit. It is abutting the medial rectus muscle. CT: computed tomography

After the fall, he began to experience headaches, which made him seek medical care. Physical examination was unremarkable. The patient had stable vital signs, a steady gait, no focal neurological deficits, and normal, symmetrical motor and sensory functions. Ocular examination revealed normal eye movements without any spontaneous or positional nystagmus. A CT scan confirmed a left subdural hygroma (Figure 1) and an incidental left anterior ethmoid air cell opacification showing a bulgy appearance that is abutting the medial rectus muscle (Figure 2). The patient refused surgery at that time.

**Figure 3:**
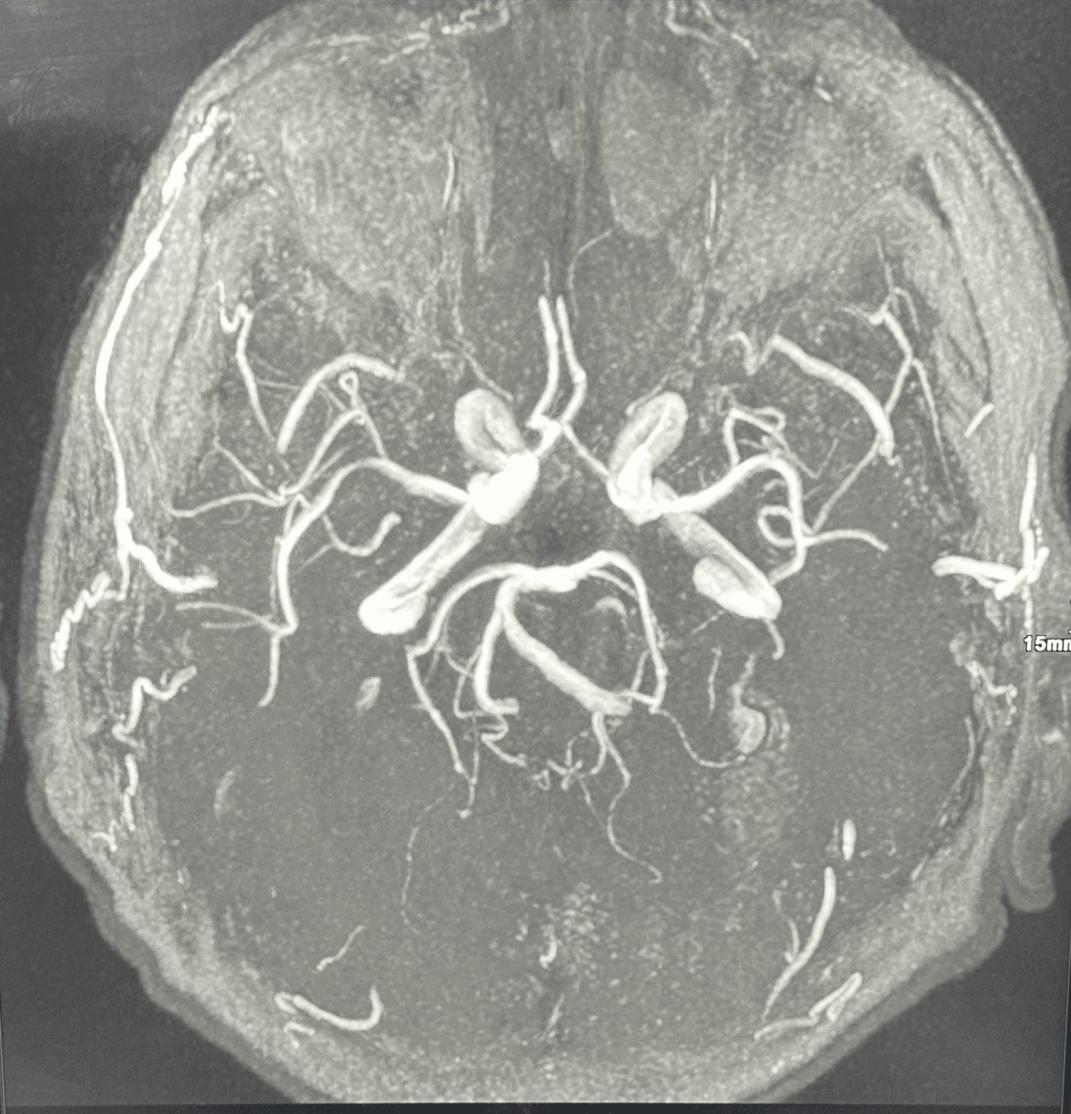
Preoperative cerebral MRI angiography Normal caliber and patency of the intracranial vessels with no significant stenosis or aneurysm. MRI: magnetic resonance imaging

**Figure 4:**
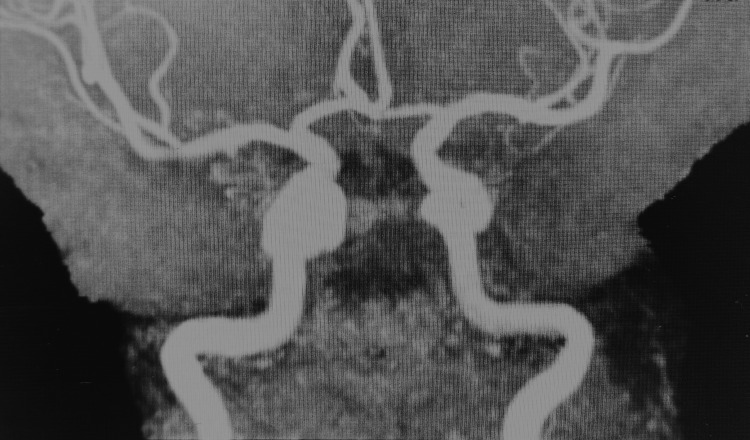
Preoperative cerebral MRI angiography Normal caliber and patency of the intracranial vessels with no significant stenosis or aneurysm. MRI: magnetic resonance imaging

**Figure 5:**
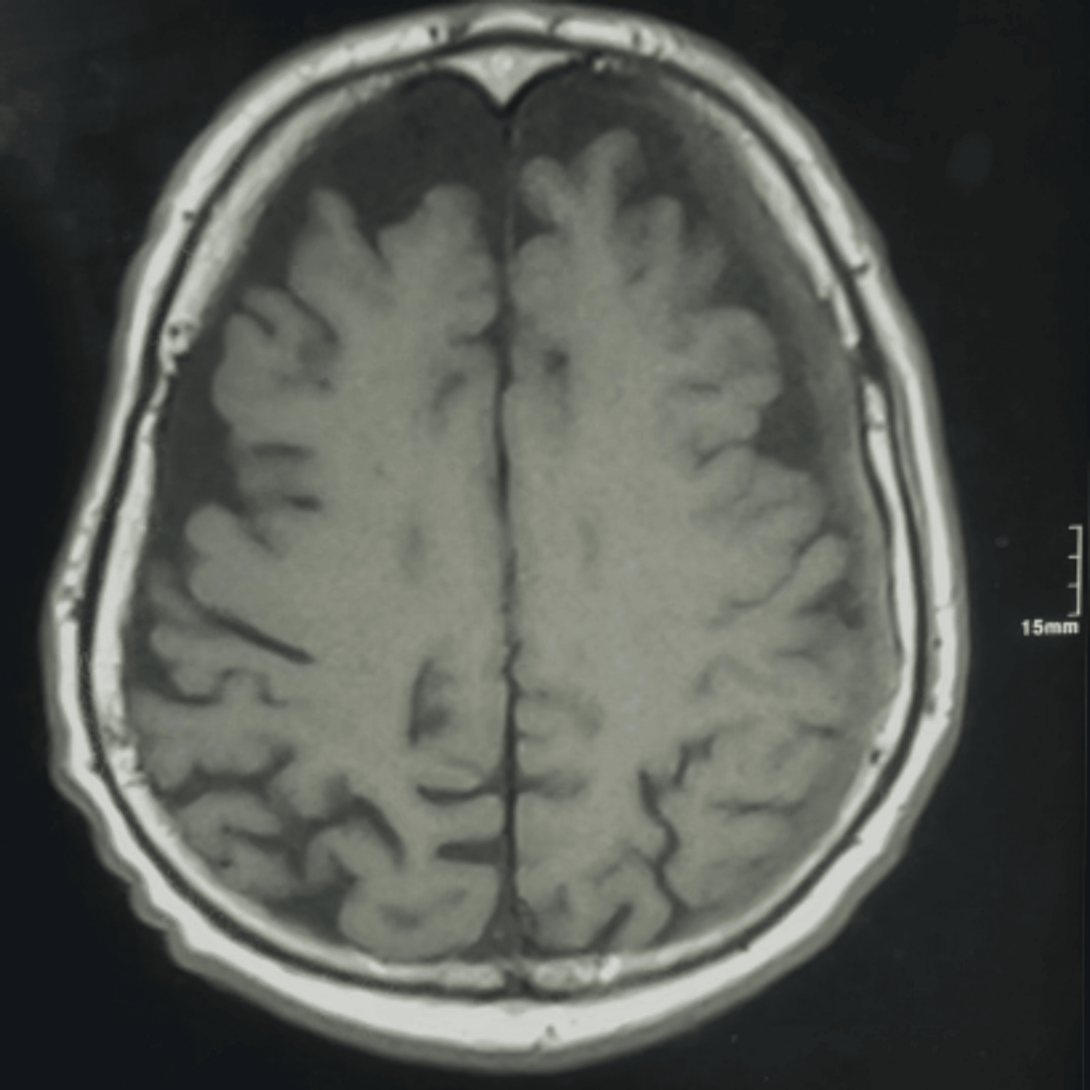
Preoperative cerebral MRI Left fronto-parietal extra-axial subdural collection suggestive of chronic subdural hematoma. MRI: magnetic resonance imaging

**Figure 6:**
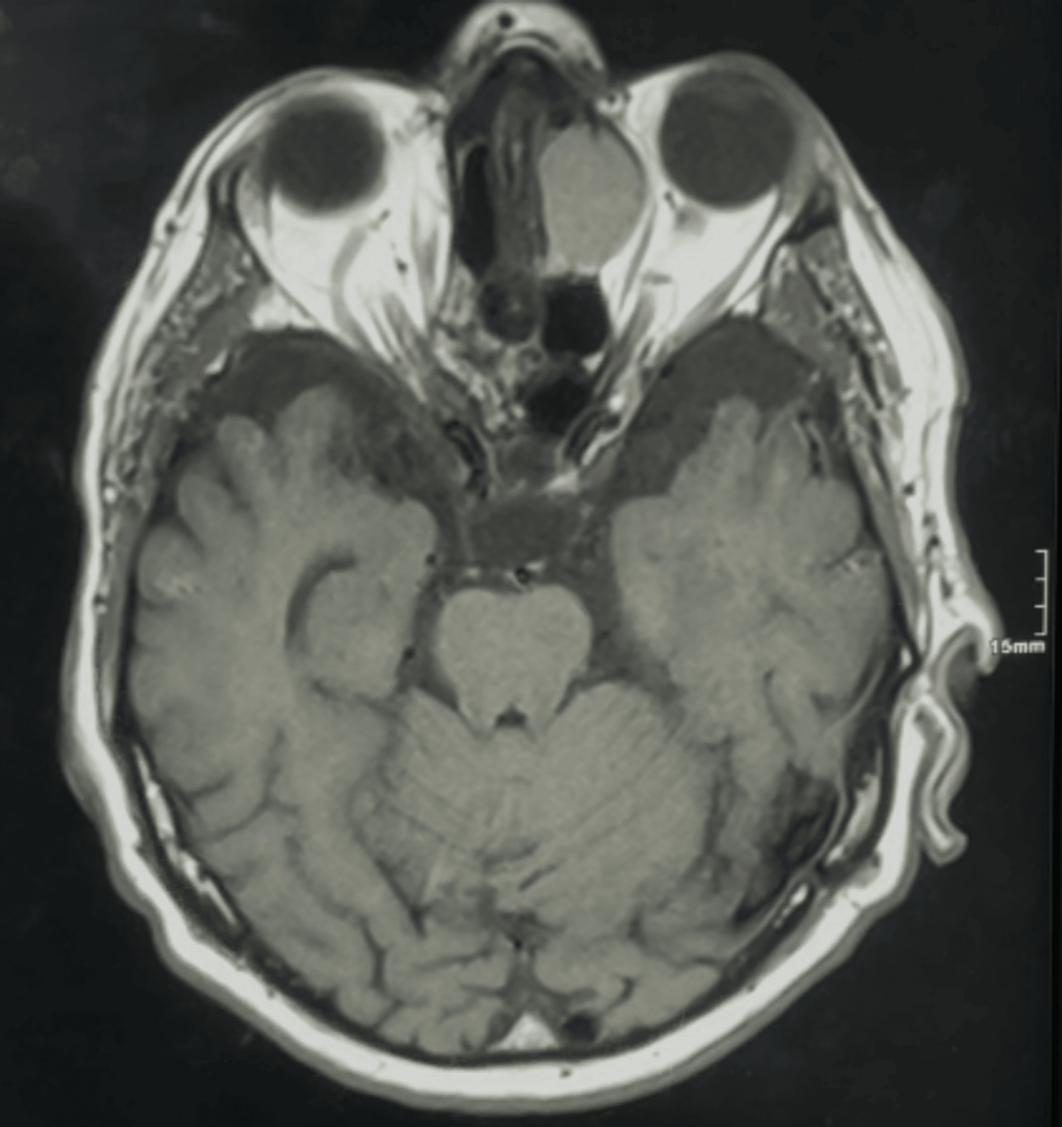
Preoperative cerebral MRI A 27 x 26 x 19 mm nodular lesion centered on the left anterior ethmoid air cells, showing a bulgy appearance with underlying bone lysis and compressing over the medial wall of the left orbit, and abutting the medial rectus muscle with no evidence of detectable neoplastic lesion without IV contrast. MRI: magnetic resonance imaging, IV: intravenous

**Figure 7:**
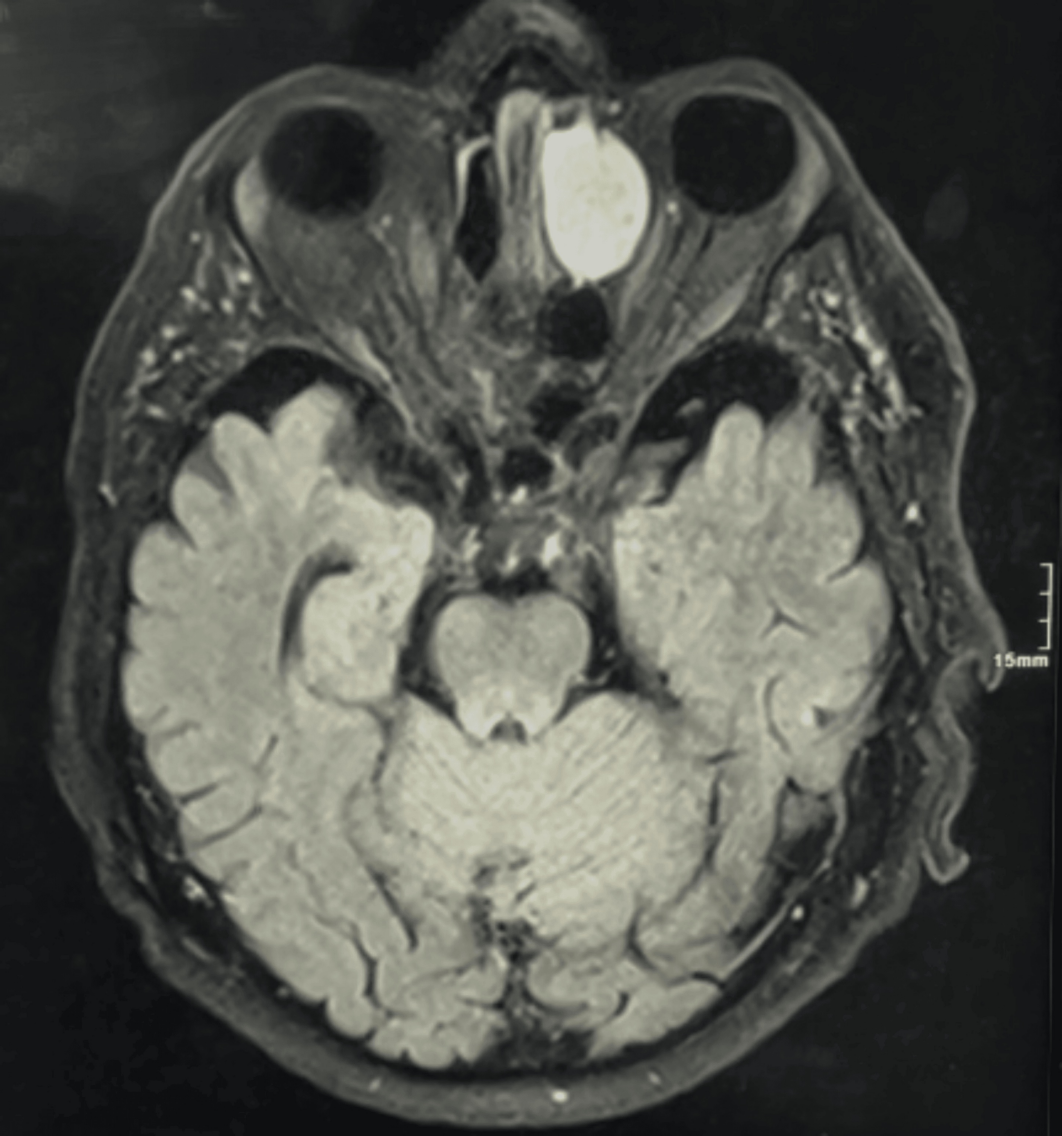
Preoperative cerebral MRI A 27 x 26 x 19 mm nodular lesion centered on the left anterior ethmoid air cells, showing a bulgy appearance with underlying bone lysis and compressing over the medial wall of the left orbit, and abutting the medial rectus muscle with no evidence of detectable neoplastic lesion with IV contrast. MRI: magnetic resonance imaging, IV: intravenous

**Figure 8:**
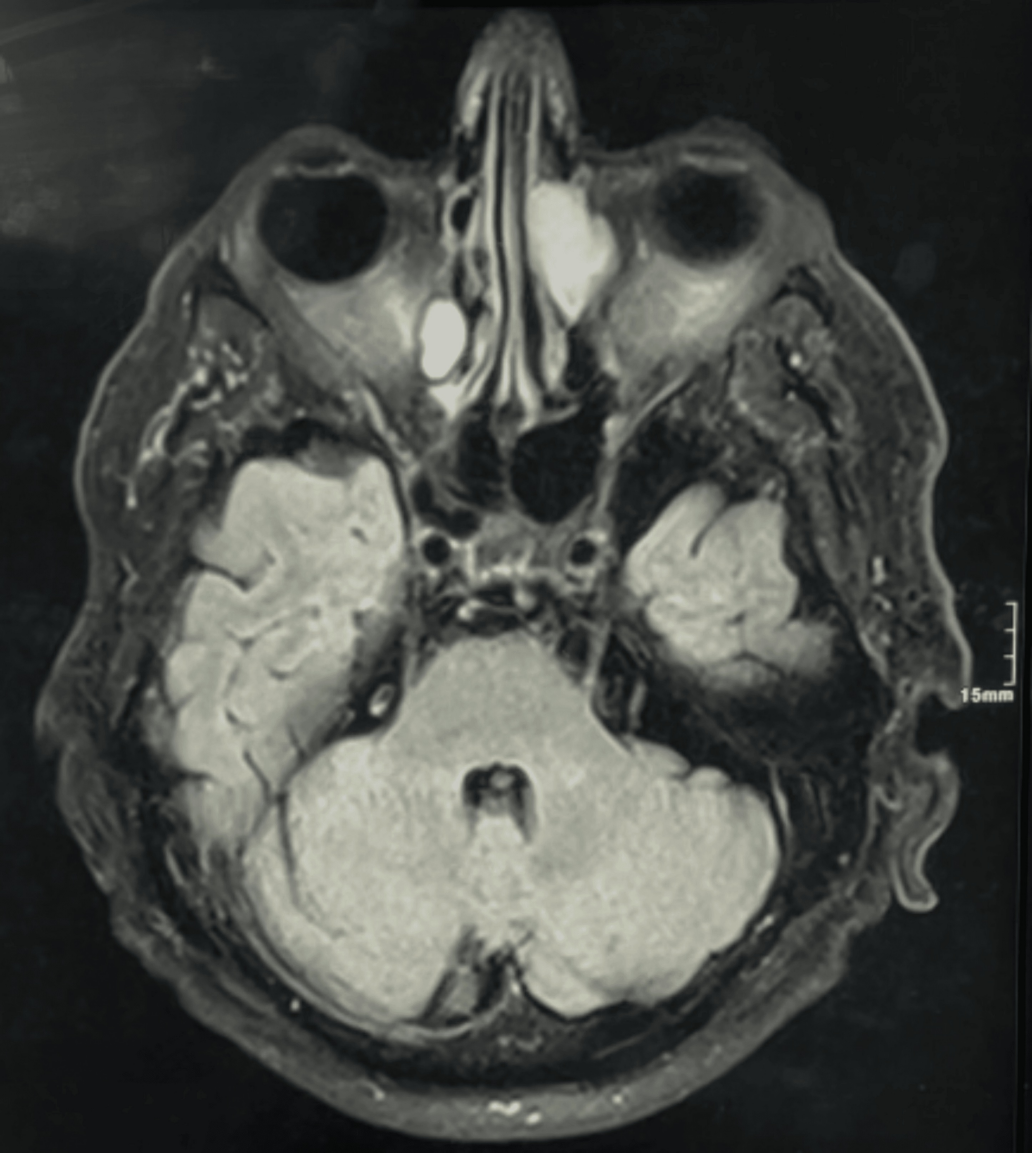
Preoperative cerebral MRI A 14 x 9 mm nodular filling of the adjacent right lamina papyracea. MRI: magnetic resonance imaging

The patient’s symptoms worsened several days later, so an MRI of the brain and an MRA were ordered. MRA did not show any abnormalities (Figures 3-4), while MRI showed features of left chronic subdural hematoma (Figure 5) and a 27 x 26 x 19 mm nodular lesion centered on the left anterior ethmoid air cells with a bulgy appearance and underlying bone lysis, also compressing over the medial wall of the left orbit (Figures 6-7). In addition, there was a 14 x 9 mm nodular filling of the right posterior ethmoid air cells, which is causing thinning of the adjacent right lamina papyracea (Figure 8), which appears similar to the previously described lesion.

**Figure 9:**
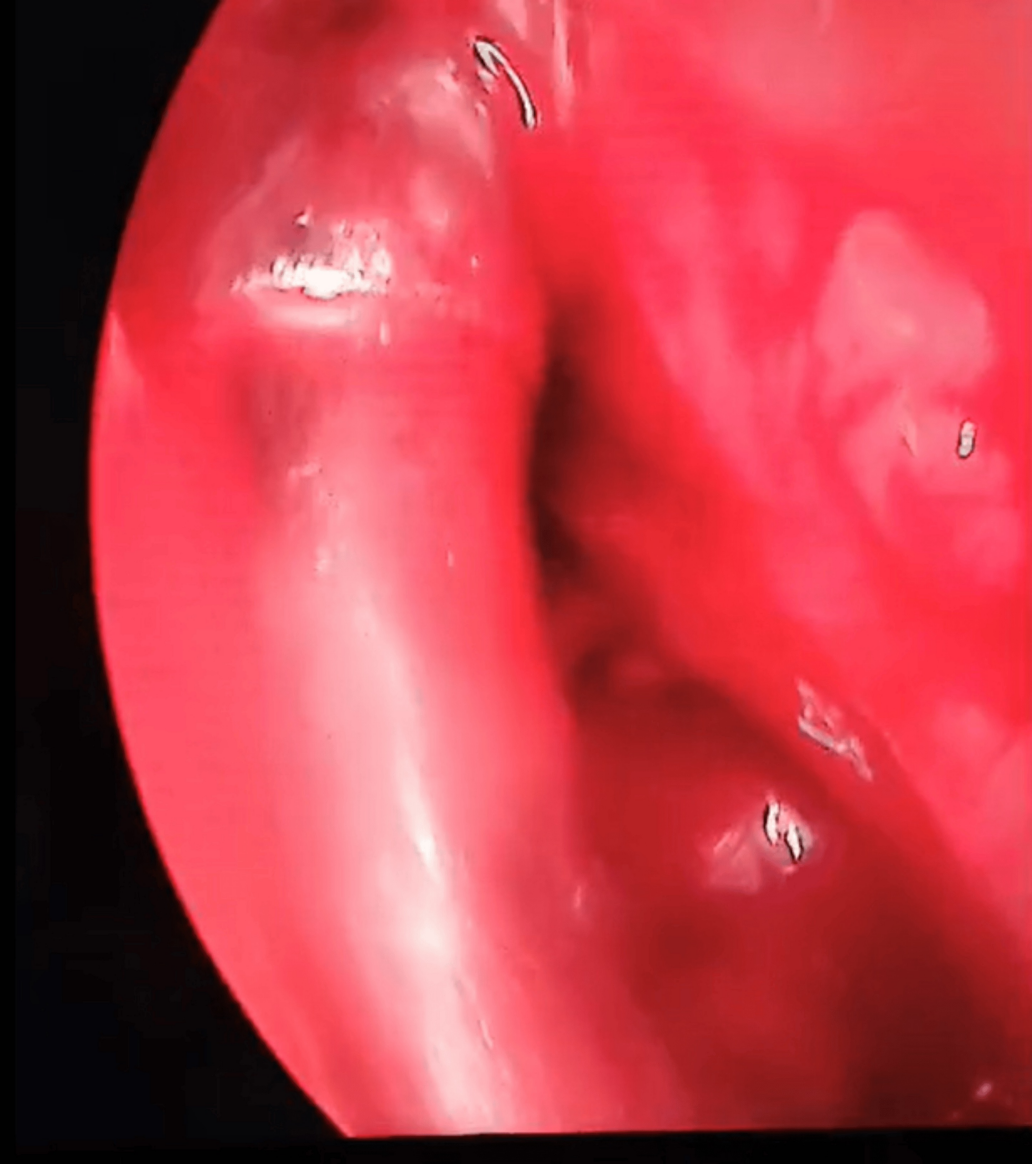
Endoscopic sinus surgery Uncapping the sinus mucocele.

**Figure 10:**
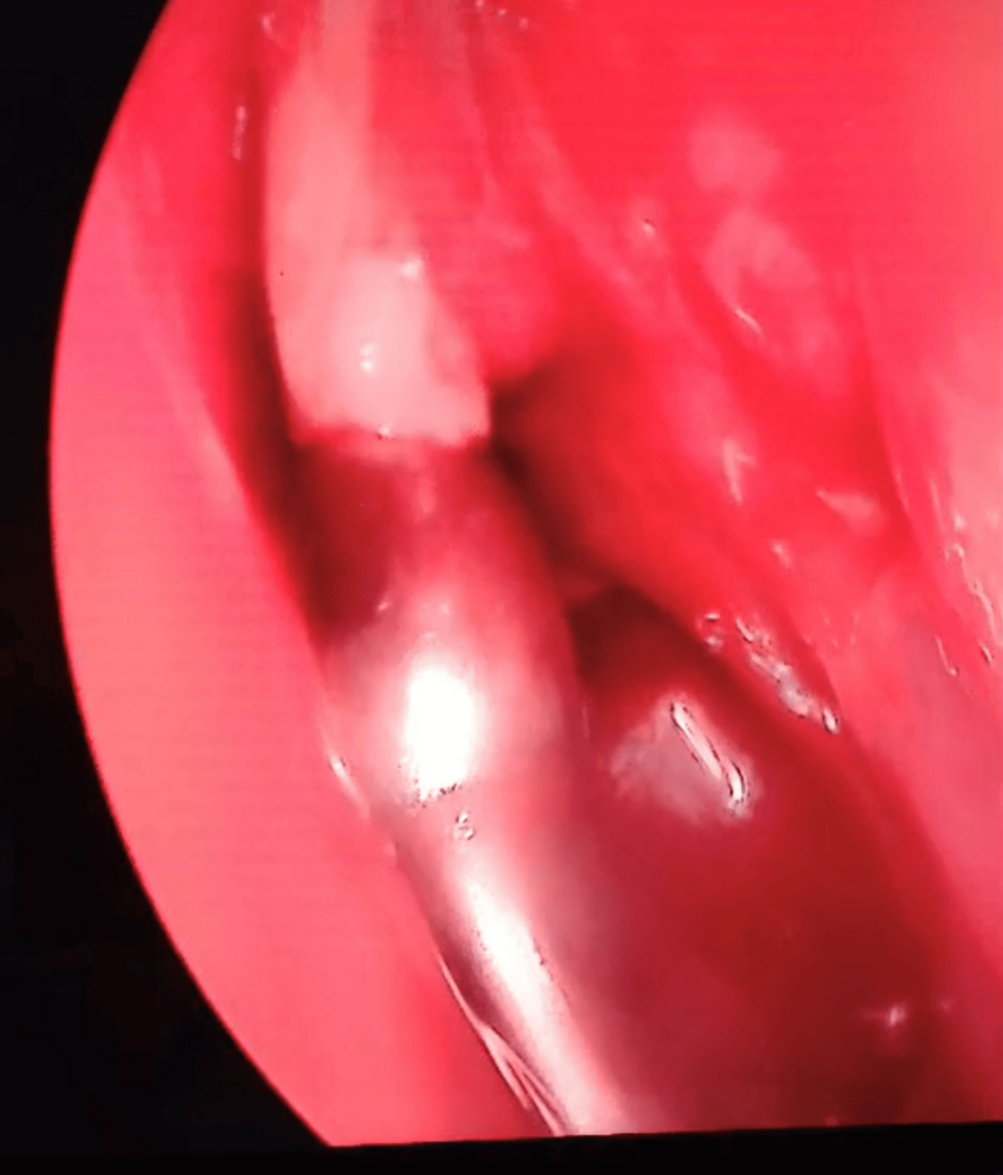
Endoscopic sinus surgery Whitish thick pus escaping the mucocele cavity and was suctioned.

**Figure 11:**
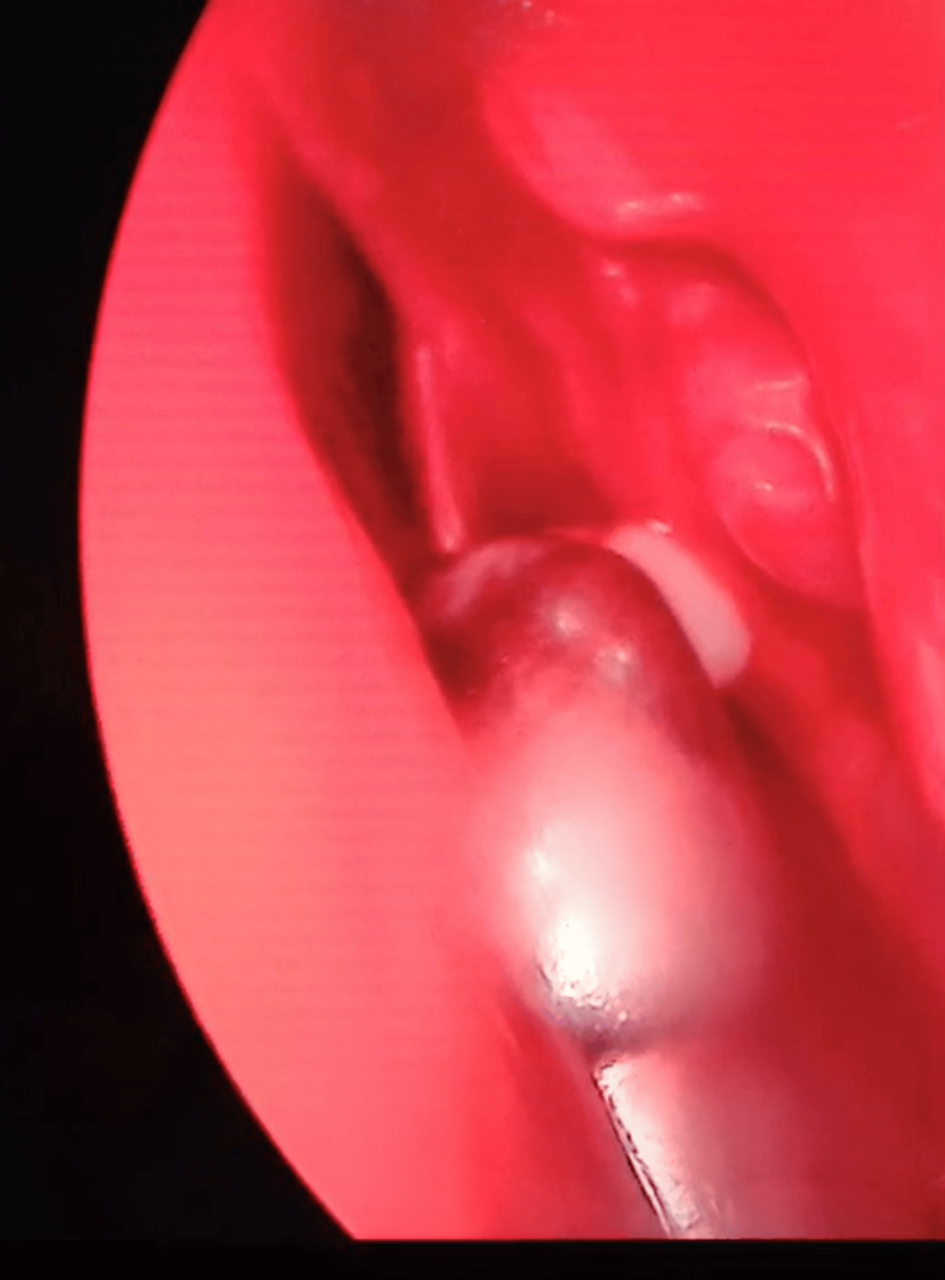
Endoscopic sinus surgery The mucocele shrinking post-draining.

The patient was admitted and was given prophylactically 2 grams of intravenous cefazolin once and 40 milligrams of intravenous methylprednisolone once, and both were administered thirty minutes prior to surgery. He did endoscopic sinus surgery and combined sinusotomies. There was a left ethmoido-frontal sinus mucocele that was abutting the left orbit. It was uncapped, and a whitish, thick pus escaped from the mucocele cavity that was suctioned (Figures 9-11). The patient’s symptoms improved rapidly and immediately following the surgery. However, the patient did not do a follow-up visit to assess his status.

## Discussion

Mucoceles are benign, expansile, mucus-filled cystic lesions that occur mainly in the paranasal sinuses [1]. They commonly arise in the frontal sinus but can also be seen in the ethmoid sinus [5]. These mucoceles are slow-growing but have the potential to become locally aggressive due to their ability to erode bone and displace adjacent structures [8]. Ethmoid mucoceles are classified based on their location as anterior or posterior. They present mainly with orbital complications due to their proximity to the optic nerve and base of the skull [9]. However, these complications occur more frequently in anterior than posterior ethmoid mucoceles because they are more likely to erode the lamina papyracea [6,10]. The most likely presentation can be divided into visual symptoms (diplopia, proptosis, blurred vision) due to direct compression of the medial orbit [10]; nasal symptoms (obstruction, discharge, facial pain) due to possible extension into the nasal cavity [11]; and rarely, complications such as intracranial extension [12,13] or orbital cellulitis [14].

To our knowledge, isolated dizziness as a presenting symptom for ethmoid mucocele has not been previously reported. In our case, the patient experienced acute-onset dizziness that triggered his fall, which persisted for several days thereafter. The absence of positional triggers, the chronicity, and the timing of onset concerning the mucocele and the subdural hematoma make the mucocele a possible contributory factor to the vestibular symptoms. It is crucial to note that the dizziness persisted in the patient after the first CT scan, despite confirming that the subdural hematoma did not worsen and remained stable several days later by MRI. This suggests that the subdural bleed may not solely explain the dizziness, and the anatomical location of the mucocele, combined with the absence of other likely causes, supports a possible causative role.

Because the exact mechanism remains speculative, these are possible explanations that associate dizziness with paranasal pathology: First, the mucocele may have a mass effect on the vestibular system indirectly through the orbital-apical pressure, which is seen in this case by the compression of the medial wall of the left orbit. Second, there might be an associated inflammation that extends to the skull base or inner ear. Third, the CNS is involved, which might be exacerbated in this case by the subdural hematoma after the patient fell. Last, another possible explanation involves trigeminal-vestibular interactions. The trigeminal nerve innervates the paranasal sinuses, including the ethmoid sinus [15]. It is also reported that the trigeminal nerve caudalis nucleus has reciprocal connections with the vestibular nuclei at the level of the brainstem [16]. Therefore, any irritation or inflammatory stimulation of the trigeminal system secondary to an anterior ethmoid mucocele may influence vestibular processing pathways, leading to vestibular symptoms such as dizziness.

## Conclusions

Considering that paranasal mucoceles are located near the orbit and the base of the skull, they are capable of presenting some unusual signs and symptoms other than those related to the sinonasal region. Some of these unusual signs and symptoms may pertain to the vestibular system. This case demonstrates the striking singular presentation of dizziness with an anterior ethmoid mucocele while reiterating that the vestibular system symptoms require broad differential considerations. Knowing this unusual association helps clinicians avoid misdiagnosis and enables timely and appropriate medical management.
